# MALDI-Mass Spectrometry Imaging to Investigate Lipid and Bile Acid Modifications Caused by Lentil Extract Used as a Potential Hypocholesterolemic Treatment

**DOI:** 10.1007/s13361-019-02265-9

**Published:** 2019-08-05

**Authors:** Michele Genangeli, Annemarie M. M. Heijens, Alice Rustichelli, Noortje Dien Schuit, Maria Vittoria Micioni Di Bonaventura, Carlo Cifani, Sauro Vittori, Tiffany Porta Siegel, Ron M.A. Heeren

**Affiliations:** 1 The Maastricht MultiModal Molecular Imaging Institute (M4I), Division of Imaging Mass Spectrometry, Maastricht University, Maastricht, The Netherlands; 2 School of Pharmacy, Chemistry unit, University of Camerino, Camerino, Italy; 3 School of Pharmacy, Pharmacology Unit, University of Camerino, Camerino, Italy

**Keywords:** Imaging mass spectrometry, Neutraceutics, Lentils, Lipids, LCMS, MALDI-MSI

## Abstract

This paper reports matrix-assisted laser desorption/ionization mass spectrometry imaging to investigate systematic effects of a lentil extract treatment to lower cholesterol levels. For this purpose, mass spectrometry imaging was used to spatially investigate modifications in the lipid composition and cholesterol levels in the brain, liver, and intestines as well as bile acids in the liver and intestine of rats treated with lentil extract. Neither the lipid composition nor cholesterol levels in the brain samples were found to be significantly different between the treated and not-treated animal groups. The hypercholesterolemic livers showed signs of steatosis (lipid marker PG 36:4), but no modifications in bile acid, cholesterol, and lipid composition. We found significant differences (AUC > 0.75) in the intestines regarding bile acid and lipid composition after treatment with the lentil extract. The treated rats showed a decreased reabsorption (increased excretion) of ursodeoxycholic acid, deoxycholic acid, and chenodeoxycholic acid and an increased deconjugation of taurine-conjugated bile acids (taurochenodeoxycholic acid, taurodeoxycholic acid, taurocholic acid, and 3-keto-taurocholic acid). This indicates that the lentil extract lowers the total cholesterol level in two synergic ways: (i) it increases the excretion of bile acids; hence, new bile acids are produced in the liver from serum cholesterol and (ii) the prebiotic effect leads to free taurine which upregulates the de novo synthesis of bile acid from cholesterol while activating LDL receptors. We demonstrate here that mass spectrometry imaging is a valuable tool for a better understanding of the effects of treatments such as for the synergistic cholesterol-lowering effect of the lentil extract.

**Electronic supplementary material:**

The online version of this article (10.1007/s13361-019-02265-9) contains supplementary material, which is available to authorized users.

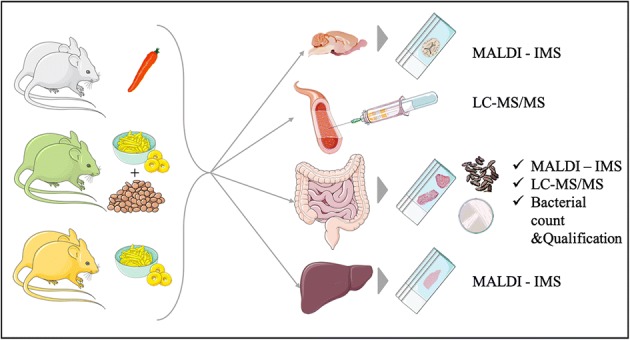

Nutrition has been known to affect a plethora of organs and functions in the body, making it an important factor in health research [1, 2]. This study aims to add to this knowledge by investigating the influence of nutrition and local cellular metabolism in the liver, intestines, and brain to provide insight into the effects of consuming vegetable extracts and phytocomplexes on the body. We focus here on lentils, which is a type of legume, high in macronutrients, mostly proteins and carbohydrates, and also rich in bioactive compounds. Regular consumption of lentils has been shown to decrease the risk for various diseases such as diabetes, obesity, cancers, and cardiovascular diseases due to the presence of bioactive compounds (e.g., polyphenols known to have health-promoting effects) [3]. The carbohydrates in lentils act as prebiotic components and are beneficial for the growth of healthy microbiota [4]. A recent publication from Micioni Di Bonaventura et al. investigating the effect of lentil extract (LE) on the cholesterol levels in rats via high-performance liquid chromatography-tandem mass spectrometry (HPLC-MS/MS) [5] concluded that a LE (*Lens culinaris* Medik) treatment lowered the total plasma cholesterol and LDL levels due to an increase in the excretion of fecal bile acids [5, 6]. This cholesterol-lowering effect was a result of the inhibition of intestinal reabsorption of bile acids caused by an active phytochemical in lentils called soyasaponin [5, 7, 8]. There are two groups of soyasaponins known as groups A and B; group B contains compounds, e.g., soyasaponin I and βg, which are mainly responsible for lowering cholesterol [5, 9].

Bile acids are produced by hepatocytes from precursor cholesterol and secreted via the bile duct into the duodenum [10, 11]. Most bile acids (around 95%) are reabsorbed in the ileum and transported back to the liver via the portal vein (the enterohepatic circulatory system) [10]. Primary bile acids are produced in the hepatocytes of the liver and then converted into secondary bile acids by the intestine microbiota. The primary bile acids, mainly cholic acid (CA) and chenodeoxycholic acid (CDCA), are conjugated with taurine and glycine to be excreted as bile into the duodenum [12]. In the intestines, the microbiota converts CA into deoxycholic acid (DCA) and CDCA into lithocholic acid (LCA) and ursodeoxycholic acid (UDCA), which are secondary bile acids [12, 13]. Because soyasaponins have an inhibiting effect on the reabsorption of bile acids, it is of great interest to investigate the metabolic modifications of bile acid and lipid compositions in the intestines after treatment with soyasaponin [5].

It was hypothesized that the changes in the intestine after LE treatment [5] could also result from altered bile acid synthesis and modifications in the lipidic composition in the liver. Additionally, we speculated that the gut–brain connection and the hypercholesterolemia altered the expression of lipids and cholesterol levels in the brain. Here, we employed the matrix-assisted laser desorption/ionization mass spectrometry imaging (MALDI-MSI) to analyze organs harvested from the same animals sacrificed in the paper from Micioni Di Bonaventura et al. [5] (Figure 1), and investigate whether LE food supplementation affects cholesterol and lipid levels of the brain, intestines, and liver, and bile acid levels in the intestines and liver [14–17]. MALDI-MSI has been proven to be an extremely valuable tool able to study the spatial distribution of a wide variety of analytes (e.g., lipid, protein, peptide, metabolites) with high sensitivity [18–20]. Compared with conventional imaging techniques such as PET/CT or MRI, MALDI-MSI offers numerous advantages: it is a label-free technique that can produce higher spatial resolution images and allows the simultaneous detection and identification of thousands of different compounds in a single experiment [21]. Additionally, when compared with other mass spectrometry techniques such as HPLC-MS/MS, MALDI-MS allows the analysis and visualization of selected molecules while maintaining their spatial distribution and the integrity of the sample.

We discovered that LE has a duplex effect in which it lowers the total cholesterol and LDL levels, where *N*-arachidonoyl taurine and taurine-conjugated BA play an important role in these effects. Furthermore, our findings show that LE does not interfere with brain and liver functionality.

## Materials and Methods

### Chemicals

Methanol, ethanol, *n*-hexane, and xylene (ULC/MS-CC/SFC grade) were purchased from Biosolve Chimie (Dieuze, France). Red phosphorous standard, acetone, Entellan, and norhamane were purchased from Merck KGaA (Darmstadt, Germany).

### Animals and Organ Collection

**Figure 1:**
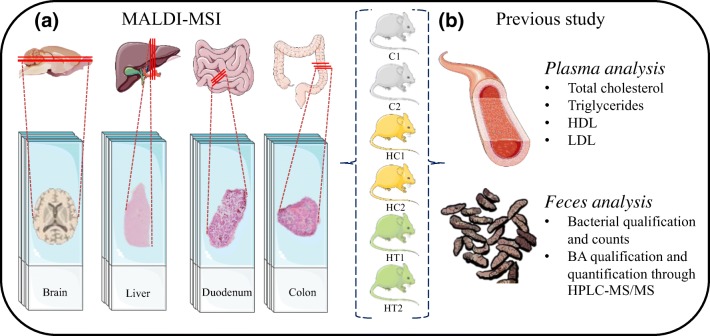
(**a**) Experimental design. A total of six rats (2× control [C], 2× hypercholesterolemic control [HC], and 2× hypercholesterolemic treated [HT]) were sacrificed. Four organs were collected from each rat (brain, liver, duodenum, and colon), sliced, and analyzed in triplicate. (**b**) The animals included in this paper are the same rats used for the paper from Micioni Di Bonaventura et al., where the listed analyses were performed. The images are meant for illustrative and explanatory purposes only. Images of the brain was created by Gill Brown [41]; other images were taken from SERVIER medical Art (CC) [https://smart.servier.com/]

The organs used for this study were harvested from the same animals sacrificed as described in the paper from Micioni Di Bonaventura et al. [5] (Figure 1). The brains, livers, duodena, and colons from 6 male Sprague–Dawley rats (*n* = 24) were analyzed in three technical replicates. The animals belonged to three study groups as follows:

i. Two rats were used as a control group and did not receive any treatment (C1 and C2) but were fed with standard laboratory chow and water ad libitum (4RF18, Mucedola, Settimo Milanese, Italy; 2.6 kcal/g);
ii. two rats received a commercially available high-cholesterol diet (AIN-76A rodent diet with 1% cholesterol and 0.5% cholic acid, D04082702 Research Diet, New Brunswick, USA) to cause hypercholesterolemia and also received water ad libitum (hypercholesterolemia rats: HC1 and HC2);
iii. The remaining two rats were fed with the same high-cholesterol diet and also received 4 ml of LE (*Lens culinaris* Medik) rich in soyasaponin diluted in 16 ml of water, as described elsewhere [3]. When they finished drinking the extract, free access to water was allowed (hypercholesterolemia rats with treatment: HT1 and HT2).

All rats were sacrificed after 71 days, and organs harvested. The intestines were flushed with water. The organs were stored separately in tin foil in a plastic bag and kept in a – 80 °C freezer prior to analysis.

### Preparation of Tissue Sections for MALDI-MSI Analysis

The organs were sectioned with a cryomicrotome (Leica CM 1860 UV) at 12-μm thickness in a 30–45% humidity atmosphere. The brain was transversally sectioned, the median lobe of the liver was axially sectioned, and consecutive transverse cross sections of the intestines (after the duodenal papillae for the duodenum) were also prepared. The tissue sections were mounted onto clean indium tin oxide (ITO) unpolished float glass slides (25 × 75 × 1.1 mm, Rs = 4–8 Ω, Delta Technologies LTD, Loveland, USA). The ITO glass slides were cleaned by sonicating for 6 min in subsequent baths of *n*-hexane and ethanol. Dust particles from the slides were removed using nitrogen before usage. The organs were randomly mounted onto the slides in order to minimize batch effects during analysis. Prior to analysis, the mounted tissue sections were desiccated under vacuum for 20 min. Then, 80–90 mg of norharmane (beta-carboline) MALDI matrix was dissolved in 2 ml of methanol. This solution was applied onto the slides with a sublimator device (HTX Technologies, LLC, Chapel Hill, USA) [22] at 140 °C for 180 s, and the samples were allowed to dry up in a vacuum desiccator for at least 20 min. Each plate was scanned (grayscale, 2400 dpi) to align the laser with a visual image. The plates were stored in vacuum bags in the − 80 °C freezer prior to MALDI-TOF analysis.

### MALDI-MSI Analysis

Lipid mass spectra were acquired with the Bruker RapifleX MALDI Tissuetyper (Bruker Daltonik GmbH, Bremen, Germany) where ions were generated with a Smartbeam 3D laser in M5 profile (Nd:YAG laser, *λ* = 355 nm) and data acquired at a spatial resolution of 50 μm. Experiments were performed in negative ionization mode over a mass range of *m/z* 300–1700. The laser intensity was set between 70 and 90%. External mass calibration was performed using a red phosphorus standard mixed with acetone [23]. MS/MS analysis was performed on a Synapt G2-Si (Waters, Manchester, UK) which utilizes collision-induced dissociation (CID) to fragment-isolated precursor ions in the trap cell with a collision energy of 25 eV and an isolation window of 1 Da. Exact mass measurements were performed on a Fourier transform ion cyclotron resonance (FT-ICR) MS system (solariX; Bruker Daltonik GmbH, Bremen, Germany), equipped with a 9.4-T magnet to provide further information for the elucidation of the identity of selected ion species. This is deployed to match the observed *m/z* values with the theoretical mass of the molecule under study and complements the MALDI-TOF–based MS/MS identification.

### Data Preprocessing and Analysis

The resulting data from the analysis of all organs were normalized to total ion count (TIC) using FlexImaging (version 4.1, Bruker Daltonik GmbH, Bremen, Germany). Average mass spectra from each organ were exported to mMass (http://www.mmass.org, version 5.5.0 [24]) where a Gaussian baseline correction was applied. A peak list was generated with a binning of 0.2 bandwidth and used for further data analysis. Multivariate analysis (i.e., unsupervised principal component analysis (PCA [25]) and probabilistic latent semantic analysis (pLSA [26]) were performed on the TIC-normalized spectra using SCiLS Lab software (version 2016b, Bremen, Germany). The PCA was used to find the highest variance and check the quality of the data, while the pLSA was employed to discover differences between groups and identify potential features responsible for these differences. For each PCA and pLSA, a total of 15 components were taken, and the score plots were studied to find components and *m/z* values which discriminate between groups C, HC, and HT.

The brain samples were analyzed by comparing the whole tissue section at first, and then by comparing then-selected regions of interest of the brains (i.e., prelimbic cortex, caudate putamen, cerebellum, and hippocampus (Figure 6a). These areas were chosen as previous studies showed that hypercholesterolemia could induce changes in the hippocampal phenotype and memory deficits [27, 28]. The liver samples were analyzed by comparing the whole tissue section and, for the intestines, the duodenum and the colon were analyzed independently.

A list containing peaks of interest related to bile acids, cholesterol, and steatotic liver-associated disease was created based on literature [5, 16] (Table S1). The peaks belonging to the aforementioned peak list were visually and statistically examined. The most relevant components from the PCA and LSA were further analyzed by evaluating the receiver operating characteristic (ROC) plot of every discriminative (AUC ≥ 0.75) peak obtained. Selected peaks with differences between groups or subgroups were then analyzed on a Synapt G2-Si using MS/MS in order to obtain additional information to identify these peaks and confirmed using the exact mass from the FT-ICR.

### Histological Staining

The matrix was removed from the tissue sections after MALDI-MSI analysis in a bath of 100% ethanol (2×) for 3 min and consecutively rehydrated in subsequent ethanol baths of 96% (2×) and 70% (2×), each for 2 min. The samples were stained with hematoxylin for 3 min, washed with deionized water, and stained with eosin for 30 s. The slides were subsequently washed under running tap water and placed in absolute ethanol for 1 min and fresh xylene for 30 s. Finally, the slides were coverslipped and fixed with Entellan. High-resolution optical images of the H&E-stained tissue microarrays were obtained with the MIRAX Desk scanner (Zeiss, Jena, Germany).

## Results and Discussion

### Cholesterol, Bile Acid, and Lipid Modifications in the Liver

**Figure 2:**
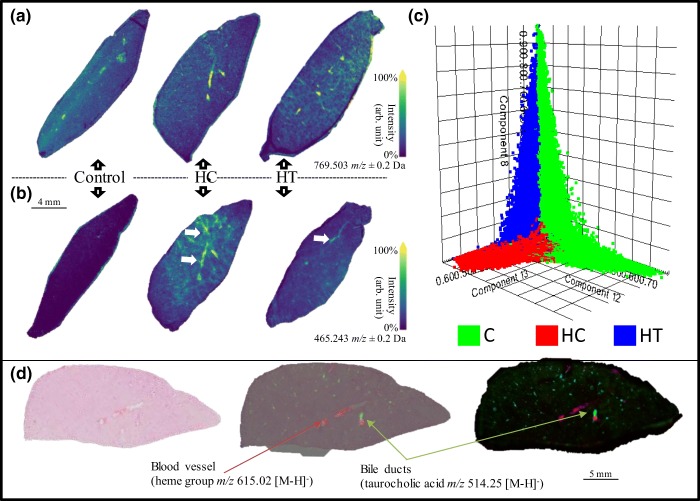
(**a**) Molecular distribution of the cholesterol sulfate. From the image, it is clear that there are no differences in regard to cholesterol expression in the three liver groups. (**b**) Molecular image of the mass peak corresponding to PG 36:4 (a marker for steatosis, as indicated by the white arrows). Results show that the signal measured for PG is relatively higher in the HC liver compared with that of the treated group and control. (**c**) Unsupervised statistical analysis (pLSA) showing a clear distribution of the three groups (green, control; red, hypercholesterolemic control; and blue, hypercholesterolemic treated). (**d**) Blood vessels and bile ducts highlighted using the heme group (*m/z* 615.02 [*M*-*H*]^−^) and the taurocholic acid (*m/z* 514.25 [*M*-*H*]^−^), respectively

In the previous study involving the same rats [5], LE was found to reduce the levels of cholesterol effect in plasma. Here, we focused our investigations on the levels of cholesterol in the liver. The control group was expected to contain the lowest cholesterol levels, followed by the hypercholesterolemic treated group, and the hypercholesterolemic control group which displayed the highest levels. In the present study however, no significant differences in cholesterol levels in the liver were found among the three groups (Figure 2a).

Next, we investigated the bile acids (BA) produced from precursor cholesterol and secreted via the bile duct and lipid levels. The bile ducts were identified by localizing and visualizing taurocholic acid ([*M*-*H*]^−^ at *m/z* 514.25) (Figure 2d). The peaks included in the generated peak list (Table S1) and related to BA and steatotic liver-associated disease were visually and statistically examined. The *m/z* values were then compared with those of the significant peaks from the pLSA. Molecular images of selected BA (Table S1) [5] did not show differences in the distribution of these molecules between the three groups. The cardiolipin at *m/z* 1465 was not detected in our samples.

With respect to lipids, pLSA of the whole liver sections displayed three components that distinguished the three different groups of treated mice (Figure 2c). From the loading plot, a total of 218 peaks contributed to this discrimination, of which 11—related to lipid species—were found to be statistically relevant (AUC > 0.75) for the discrimination between two or all three groups. These peaks were identified with MS/MS and confirmed using the exact mass from the FT-ICR (Table 1, Table S2, Figure S1). PE (18:0_20:4), PE (18:0_22:4), PI (16:0_20:4), and PI (18:0_20:4) were mainly expressed in the control group in comparison with the other two groups and they mostly contain a chain of arachidonic acid. PI with a fatty acid chain of arachidonic acid (20:4) has been shown to be connected with a healthy liver [16], in line with our observations. PE (16:0_18:2), PI (16:0_18:2), and PI (18:0_18:2) did not show noticeable differences between groups, but only differences between individual organs.

**Table 1:** List of Bile Acids, Soyasaponins, and *N*-Arachidonoyl Taurine, Confirmed with the Exact Mass. Starting from the Left Column, We Have the Compound Name, Theoretical Mass, Measured Mass, and Error Expressed in ppm from a Low-Mass Resolution (Rapiflex) and a High-Mass Resolution (Solarix) Instruments. Soyasaponins Were Not Found in the Analyzed Tissues

		Rapiflex	Rapiflex	Solarix	Solarix
Compounds [*M*-*H*]^−^	Theor. mass	Measured *m*/*z*	ppm err.	Measured *m*/*z*	ppm err.
CA	407.28029	407.3	− 48.3918	407.2805	− 0.5156
LCA	375.29046	375.3	− 25.4197	375.2909	− 1.1724
DCA	391.28538	391.2	218.2515	391.2850	0.9712
CDCA	391.28538	391.2	218.2515	391.2850	0.9712
UDCA	391.28538	391.2	218.2515	391.2850	0.9712
TDCA	498.28948	498.3	− 21.1118	498.2904	− 1.8463
TCDCA	498.28948	498.3	− 21.1118	498.2904	− 1.8463
3keto-TCA	512.26874	512.3	− 61.0189	512.2689	− 0,3123
TCA	514.28439	514.3	− 30.3519	514.2837	1.3417
*N*-Arachidonoyl taurine	410.23705	410.2	90.3218	410.2375	− 1.0969
SOYA I	941.51153	NA	NA	NA	NA
SOYA BG	1067.54323	NA	NA	NA	NA

The last four identified lipids were PG (36:4), known to be associated with steatotic livers [16], PE (40:6), PG (38:5), and PI (16:0_22:6). These four lipids displayed higher levels in the HC group (Figure 2b).

The cholesterol and BA expression in the liver were statistically relevant for discrimination between groups. On the other hand, the C groups showed a higher expression in PI with a fatty acid chain of arachidonic acid (20:4), fatty acid associated with healthy liver [16]. However, we showed that the HC group showed higher signals of a steatosis-associated lipid.

### Cholesterol, lipid, and bile acid modifications in the intestine (colon and duodenum)

**Figure 3:**
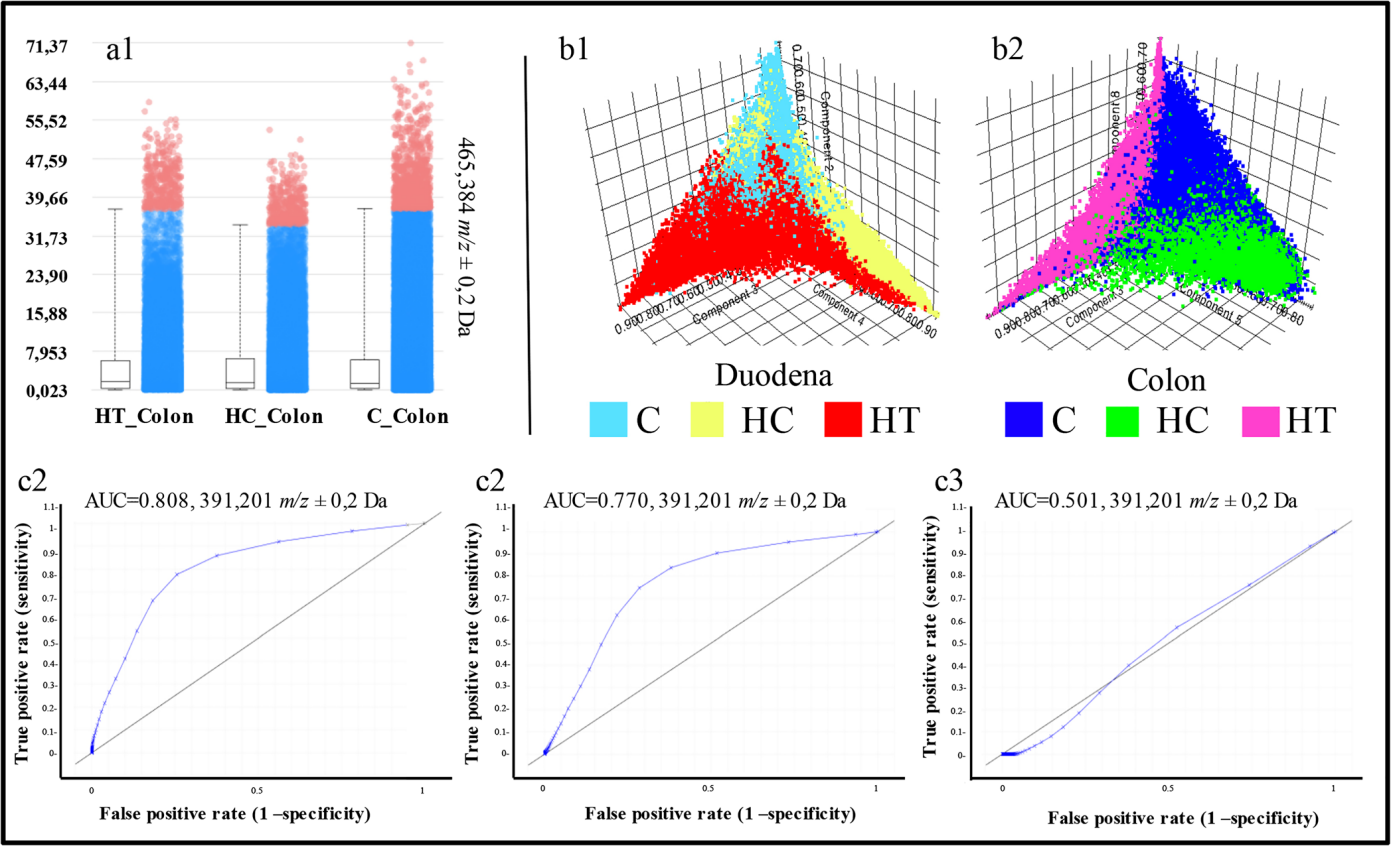
(**a**) Cholesterol level in the three colon groups. No significant variations are shown. (**b1, b2**) Score plot from the pLSA between the three duodena groups (**b1,** C, light blue; HC, yellow; HT, red) and between the three colon groups (**b2**, C, blue; HC, green; HT, pink). Both the pLSA show differences between the groups, especially in the colon. (**c1–c3**) ROC plots for peak *m/z* 391.2 (CDCA/DCA/UDCA) which show significant differences in the expression of these BA in the (**c1**) HT_Colon vs C_Colon and (**c2**) HT_Colon vs HC_Colon and no differences in the (**c3**) HT_Duodenum vs HC_Duodenum

Next, we examined the differences in cholesterol levels in the intestines. In negative ionization mode, cholesterol can be detected as cholesterol sulfate, which is typically detected at *m/z* 465 and *m/z* 467 [29]. Both of these peaks were present in the obtained spectra, but they were not discriminating between the groups. Their distribution was similar for both the colon and the duodenum (Figure 3a).

With respect to the lipid composition in the intestines, no significant differences were observed between groups (data not shown). A possible reason for this is that the LE treatment rich in soyasaponins only has an effect on the bile acid composition via the microbiota and might not directly affect the lipid composition. We investigated the presence of soyasaponin I and βg, the cholesterol-lowering agents found in lentils, with *m/z* 941.5 and *m/z* 1067.0, respectively [30]. Additionally, peaks related to the conjugation of soyasaponins and bile acids were also examined. However, no peaks were found, indicating that no soyasaponins could be detected in the intestines.

Even though we did not find differences in the cholesterol and lipid distribution in the intestine and did not detect any soyasaponins, the PCA showed a different variance within both the duodenum (*n* = 3) and colon (*n* = 3) groups, analyzed independently (Figure 3b). The HT and HC groups showed a clear separation, both overlapping the control group (Figure 3b). We identified a total of 58 *m/z* values able to discriminate between HT and HC groups both in the duodenum and colon with an AUC ≥ 0.75. Comparing these 58 peaks with the list of bile acids quantified from Micioni Di Bonaventura et al. [5], we identified chenodeoxycholic acid, deoxycholic acid, and ursodeoxycholic acid (UDCA, DCA, CDCA, *m/z* 391.2) to be significantly higher in the colon but not duodena of the HT group compared with the HC rats (Figure 3c). This observation suggests that the reuptake of these bile acids is inhibited by the LE diet, likely by soyasaponins. However, lithocholic acid (LCA, *m/z* 375.3) and cholic acid (CA, *m/z* 407.0) do not discriminate between the groups.

**Figure 4:**
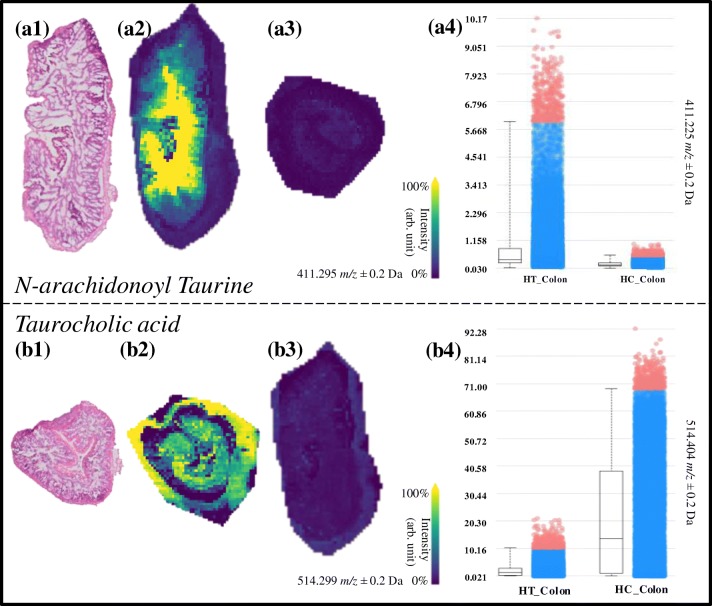
(**a1–a4**) Molecular images of *N*-arachidonoyl taurine in the HT_Colon (**a2**) and HC_Colon (**a3**). (**a4**) Boxplot of the intensity of *N*-arachidonoyl taurine in the HT_Colon vs. the HC_Colon. (**b1–b4)** Molecular images of taurocholic acid in the HC_Colon (**b2**) and HT_Colon (**b3**). (**b4**) Boxplot of the intensity of *N*-arachidonoyl taurine in the HT_Colon vs. the HC_Colon

Taurodeoxycholic acid and/or taurochenodeoxycholic acid (TDCA, TCDCA, *m/z* 498.3), 3-keto-taurocholic acid (3keto-TCA, *m/z* 512.3), and taurocholic acid (TCA, *m/z* 514.3) were all discriminative (AUC ≥ 0.748) between HC_Colon and HT_Colon, with higher levels in control animals (Figure 4). These bile acids were also discriminative between the colon and duodenum of the HC group, suggesting that they undergo modifications while traveling through the digestive tract. As seen in Figure 4b, taurocholic acid (*m/z* 514.3) is mainly present in the HC_Colon group. It is clear that these three molecular species that are taurine-conjugated bile acids have a strong discrimination power between HC_Colon and HT_Colon and are with a higher amount present in the HC_Colon image (Figure 4b). *N*-Arachidonoyl taurine is a polyunsaturated fatty acid (PUFA) conjugated with a taurine group that acts in the brain as a neurotransmitter and plays a role in insulin secretion in the pancreas [31, 32]. *N*-Arachidonoyl taurine is discriminative (AUC > 0.750) between HT_Colon and HC_Colon (Figure 4a), being prevalently present in the HT_Colon.

**Figure 5:**
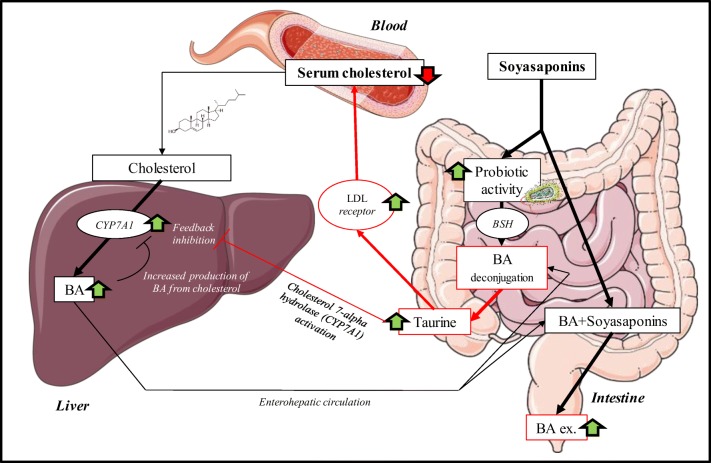
Overview of the duplex cholesterol-lowering mechanisms of soyasaponins by increasing the excretion of BA with the faces, which stimulates the production of new BA from cholesterol. Increased probiotic activity which results in an increased release of free taurine. Taurine both increases the activity of the cholesterol 7-alpha hydrolase enzyme in the liver (CYP7A1) and stimulates LDL receptors in the body, resulting in an overall reduction of serum cholesterol. The images are meant for illustrative and explanatory purposes only. Images were taken from SERVIER medical Art (CC) [23]

Deconjugation of taurine-conjugated bile acids is carried out by the gut microbiota, namely by the following bacterial genera: *Bacteroides*, *Bifidobacterium*, *Clostridium*, *Lactobacillus*, and *Listeria* [33]. Deconjugation occurs by bile salt hydrolase (BSH) enzymes, which are highly present in *Bifidobacterium* (Figure 5). The end products of this reaction are free taurine and deconjugated bile acid [34] which were detected in the present study. Our results correlate with the work of Micioni Di Bonaventura et al. who indicated an increase of the *Bifidobacterium* in the LE-treated group [5]. A previous study demonstrated that dietary taurine supplementation reduced serum cholesterol levels by increasing the activity of hepatic cholesterol 7a-hydroxylase (CYP7A1), which is involved in the process of converting cholesterol into bile acids and increases the fecal excretion of bile acids in hypercholesterolemic rats [35]. The deconjugation of the taurine-conjugated bile acids by the *Bifidobacterium* will act similarly to taurine supplementation, as more of the taurine will come free and will be reabsorbed before it reaches the colon by the taurine transporter [36] (Figure 5). Taurine also plays a role in elevating LDL receptor levels, which results in lower cholesterol serum levels [37]. When the results of the increased bile acid excretion (CDCA, DCA, and/or UDCA) and the decreased taurine-conjugated bile acids (TDCA/TCDCA, 3keto-TCA, and TCA) in the HT group are combined with the findings from Micioni di Bonaventura et al., we can conclude that the taurine-conjugated bile acids lost their taurine (TDCA/TCDCA) and that the other bile acids (DCA/CDCA) end in the feces. This can be attributed to the prebiotic activity of LE rich in soyasaponins. Although we did not detect the molecules responsible for the hypocholesterolemic effect (soyasaponin I and βg [5]), we prove here that MALDI-MSI allowed the understanding of their effects in the intestines (Figure 5).

### Investigation of Cholesterol and Lipid Modifications Induced on the Brain by the LE Treatment

**Figure 6:**
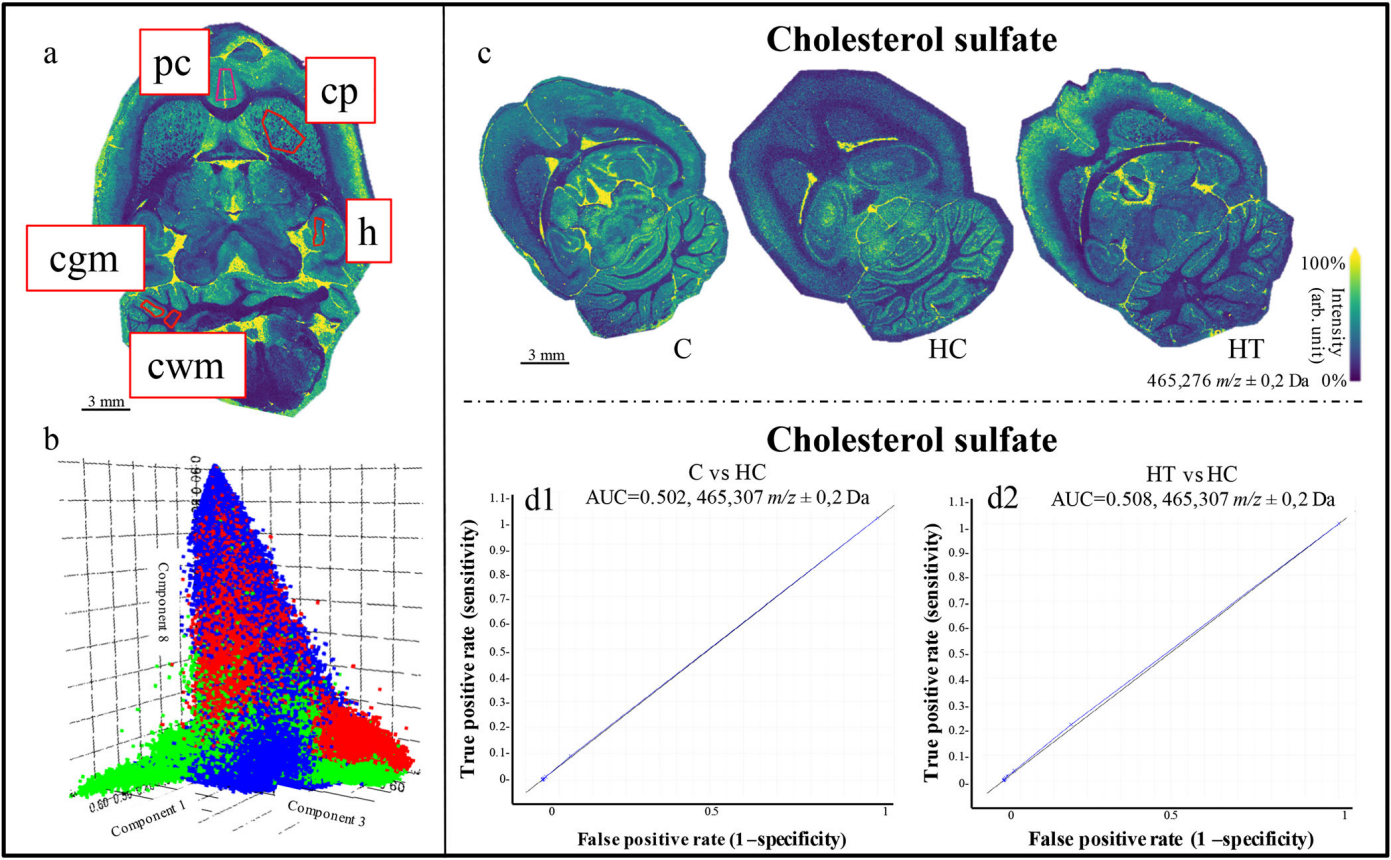
(**a**) Molecular images of the brain highlighted in red; the regions of interest (ROIs) delineated for the analysis: prelimbic cortex (pc), caudate putamen (cp), hippocampus (h), white (cwm) and gray (cgm) matter in the cerebellum. (**b**) 3D loading plot for the pLSA of the whole brains of the three groups. Blue represents the control group (C), green the hypercholesterolemic control (HC), and red the hypercholesterolemic treated group (HT). Results display no separation among the different groups. (**c**) Molecular images based on the distribution of cholesterol sulfate (*m/z* 465.3) over the whole brain for the C, HC, and HT groups. (**d1, d2**) ROC plots of the *m/z* 465.3 (cholesterol sulfate) from the comparison between (**d1**) C and HC, and between (**d2**) HT and HC

The pLSAs and PCAs for the whole brain sections and subsections of the three experimental groups (C, HC, HT) did not show statistically relevant differences (Figure 6b). Additionally, the peak related to cholesterol sulfate and connected molecular images of the distribution of cholesterol in the brains showed differences between different animals but not related to different groups. (Figure 6c, d). The caudate putamen, hippocampus, prelimbic cortex, and cerebellum (white and gray matter) were investigated due to their connection to memory deficit and possible changes in the hippocampal phenotype associated with hypercholesterolemia and obesity [27, 28]. Preliminary behavioral tests performed from Micioni Di Bonaventura et al. showed no significant behavioral change among the three experimental groups [5]. Here, no significant differences were found in cholesterol or lipid composition in these subregions. As previous studies have only shown an association between hypercholesterolemia, obesity, and memory deficits [28], it seems reasonable that there are no relevant results from the behavioral tests. These results indicate that neither the high-cholesterol diet nor LE treatment led to significant molecular variations in the brain lipid composition and distribution in rats observable with MALDI-MSI.

The absence of lipid and cholesterol changes in the brain is due to various factors. Studies showed that a high-cholesterol diet could induce relevant increases in plasma and liver cholesterol, but not brain cholesterol levels [38], in line with our observations. Additionally, the duration of the high-cholesterol diet and the treatment was too short to lead to concrete changes in brain lipid composition. This speculation is based on previous knowledge that total cholesterol absorption, synthesis, and turnover are strongly correlated with body fat composition and higher in obese subjects compared with non-obese subjects [39]. It is worth noting that the rats were sacrificed in non-obese conditions.

Furthermore, in healthy brains, the several mechanisms involved in the maintenance of brain homeostasis (e.g., apolipoprotein E, ABC transporters) actively regulate the level of cholesterol in the brain by blocking its entrance and activating or blocking secondary pathways for cholesterol synthesis [40]. This led to conclude that the hypercholesterolemic treatment did not affect the brain lipid composition and distribution within the timeframe of the study.

## Conclusions

The present study employed MALDI-MSI to investigate modifications in cholesterol, lipid, and bile acid metabolism in rat liver and intestine as well as cholesterol and lipid composition in rat brain for a better understanding of the effect of a treatment with a cholesterol-lowering, hydro-alcoholic LE-containing soyasaponins. We demonstrated that the LE does not modify the cholesterol and lipidic content of the brain nor the cholesterol composition in the liver. On the other hand, in the liver, we highlight the presence of a lipid (PG 36:4) connected with steatosis in untreated hypercholesterolemic rats.

The molecular tissue analysis results are found to be in line with previously reported observations by Micioni Di Bonaventura et al. demonstrating that some BA (UDCA, DCA, CDCA, *m/z* 391.2) are not reabsorbed in the intestine after LE treatment. Additionally, we discovered that the hypocholesterolemic effect of LE treatment rich in soyasaponins is not only due to the increased excretion of BA with the feces (which forces the animal to produce new BA from cholesterol), but also to the prebiotic effect of LE. This led to an increased hydrolization of taurine-conjugated bile acids from the gut microbiota, resulting in free taurine. Taurine has an upregulating effect on hepatic cholesterol 7a-hydroxylase and LDL receptors leading to a reduction of the total cholesterol levels in the blood.

We here demonstrated that MALDI-MSI is a valuable tool to investigate and locate several molecular changes in different tissue types leading to a better understanding of the mechanisms behind the cholesterol-lowering treatments. Specifically, an untargeted technique such as MALDI-MSI led us to the discovery of the multi-organ/synergistic biological effects of LE providing a better insight into its cholesterol-lowering properties in rat models.

## Electronic Supplementary Material

ESM 1 (DOCX 70.1 kb)
