# Supplementary material for: MALDI-Mass Spectrometry Imaging to Investigate Lipid and Bile Acid Modifications Caused by Lentil Extract Used as a Potential Hypocholesterolemic Treatment
Source: J Am Soc Mass Spectrom. 2019 Aug 5;30(10):2041–50. doi: 10.1007/s13361-019-02265-9 (PMC6805814; doi:10.1007/s13361-019-02265-9)
Supplement: Supplementary file 1 — (DOCX 70.1 kb) [file 13361_2019_2265_MOESM1_ESM.docx]

MALDI-mass spectrometry imaging to investigate lipid and bile acid modifications caused by lentil extract used as a potential hypocholesterolemic treatment.

Michele Genangeli^1,3^ Annemarie Heijens^1^, Alice Rustichelli^1^, Noortje Dien Schuit^1^, Maria Vittoria Micioni Di Bonaventura^2^, Carlo Cifani^2^, Sauro Vittori^3^, Tiffany Porta Siegel^1^ and Ron M.A. Heeren^1^*

1. The Maastricht MultiModal Molecular Imaging Institute (M4I), Division of Imaging Mass Spectrometry, Maastricht University, Maastricht, The Netherlands
2. School of Pharmacy, Pharmacology Unit, University of Camerino, Camerino, Italy
3. School of Pharmacy, Chemistry Unit, University of Camerino, Camerino, Italy

* Corresponding author: r.heeren@maastrichtuniversity.nl

**Table of content**

[**Table S1** - Peak list generated from the literature containing expected peaks with relative m/z values in negative ion mode [5,17]. 2](#_Toc8647105)

[**Table S2** - Lipid identification. Prec. = Precursor ion, Theor. mass = Theoretical mass (m/z) 3](#_Toc8647106)

[**Figure S1** - Loading plot from the major components of the pLSA in the range m/z 600-1050 for the liver groups (C, HC, HT). The identified lipids described in this paper are highlighted. Component 4 is mainly representative for the Heme group. Component 11 is mainly representative for PE(16:0_18:2), PE(18:0_20:4), PG36:4, PE(18:0_22:4). Component 12 and 13 are mainly representative from all the PI. 4](#_Toc1985039)

**Table S1** - Peak list generated from the literature containing expected peaks with relative m/z values in negative ion mode [5,17].

| **Molecule** | ***m/z* [M-H]^-^** |
| --- | --- |
| Lithocholic acid (LCA) | 375.57 |
| Cholesterol | 385.65 |
| Deoxycholic acid (DCA) | 391.58 |
| Chenodeoxycholic acid (CDCA) | 391.58 |
| Ursodeoxycholic acid (UDCA) | 391.58 |
| Hyodeoxycholic acid (HDC) | 391.58 |
| Cholic acid (CA) | 407.57 |
| Glycochenodeoxycholic acid (GCDC) | 448.62 |
| Glycocholic acid (GC) | 464.63 |
| Taurochenodeoxycholic acid (TCDCA) | 498.71 |
| Taurocholic acid (TCA) | 514.71 |
| PA (18:2_18:2) | 695.47 |
| PA (18:1_18:2) | 697.48 |
| PE (16:0_18:2) | 714.51 |
| PE (18:0_18:2) | 742.54 |
| PE (18:0_20:4) | 766.54 |
| PE (18:0_22:4) | 794.57 |
| PI (16:9_18:2) | 833.52 |
| PI (16:0_20:4) | 857.52 |
| PI (18:0_18:2) | 861.55 |
| PI (18:0_20:4) | 885.55 |
| Cardiolipin | 1465.00 |

**Table S2** - Lipid identification. Prec. = Precursor ion, Theor. mass Theoretical mass (m/z)

|  |  | **Solarix** | **Solarix** | **Synapt** | **Synapt** | **Synapt** |  |  |  |
| --- | --- | --- | --- | --- | --- | --- | --- | --- | --- |
| **Prec.** | **Theor. mass** | **measured *m/z*** | **ppm err.** | **Prod. ion -measured** | **Prod. ion theor.** | **ppm err.** | **Prod. ion id.** | **name** | **Adduct** |
| 714,51 | 714,507929 | 714,5078 | 0,18054 | 279,23 | 279,232900 | 10,38570 | FA 18:2 | PE (16:0_18:2) | [M-H]- |
|  |  |  |  | 255,23 | 255,232954 | 11,57387 | FA 16:0(+O) |  |  |
|  |  |  |  |  |  |  |  |  |  |
| 833,52 | 833,518553 | 833,5183 | 0,30353 | 279,23 | 279,232900 | 10,38570 | FA 18:2 | PI (16:0_18:2) | [M-H]- |
|  |  |  |  | 255,23 | 255,232954 | 11,57387 | FA 16:0(+O) |  |  |
|  |  |  |  | 259,01 | 259,022443 | 48,04062 | PI(259) |  |  |
|  |  |  |  | 241,00 | 241,011878 | 49,28631 | PI(241) |  |  |
|  |  |  |  | 223,00 | 223,001313 | 5,88789 | PI(223) |  |  |
|  |  |  |  |  |  |  |  |  |  |
| 861,55 | 861,549853 | 861,5499 | -0,05455 | 283,26 | 283,264254 | 15,01800 | FA 18:0(+O) | PI (18:0_18:2) | [M-H]- |
|  |  |  |  | 279,26 | 279,232900 | -97,04218 | FA 18:2 |  |  |
|  |  |  |  | 259,01 | 259,022443 | 48,04062 | PI(259) |  |  |
|  |  |  |  | 241,00 | 241,011878 | 49,28631 | PI(241) |  |  |
|  |  |  |  | 223,00 | 223,001313 | 5,88789 | PI(223) |  |  |
|  |  |  |  |  |  |  |  |  |  |
| 766,54 | 766,539229 | 766,5393 | -0,09262 | 303,22 | 303,232954 | 42,72146 | FA 20:4(+O) | PE (18:0_20:4) | [M-H]- |
|  |  |  |  | 283,26 | 283,264254 | 15,01800 | FA 18:0(+O) |  |  |
|  |  |  |  |  |  |  |  |  |  |
|  |  |  |  |  |  |  |  |  |  |
| 794,57 | 794,570529 | 794,5707 | -0,21521 | 480,29 | 480,309563 | 40,73164 | -FA 22:4(-H) | PE (18:0_22:4) | [M-H]- |
|  |  |  |  | 283,26 | 283,264254 | 15,01800 | FA 18:0(+O) |  |  |
|  |  |  |  |  |  |  |  |  |  |
|  |  |  |  |  |  |  |  |  |  |
| 857,52 | 857,518553 | 857,5186 | -0,05481 | 303,22 | 303,232954 | 42,72146 | FA 20:4(+O) | PI (16:0_20:4) | [M-H]- |
|  |  |  |  | 259,01 | 259,022443 | 48,04062 | PI(259) |  |  |
|  |  |  |  | 255,23 | 255,232954 | 11,57387 | FA 16:0(+O) |  |  |
|  |  |  |  | 241,00 | 241,011878 | 49,28631 | PI(241) |  |  |
|  |  |  |  | 223,00 | 223,001313 | 5,88789 | PI(223) |  |  |
|  |  |  |  |  |  |  |  |  |  |
| 885,55 | 885,549853 | 885,5499 | -0,05307 | 303,22 | 303,232954 | 42,72146 | FA 20:4(+O) | PI (18:0_20:4) | [M-H]- |
|  |  |  |  | 283,26 | 283,264254 | 15,01800 | FA 18:0(+O) |  |  |
|  |  |  |  |  |  |  |  |  |  |
|  |  |  |  |  |  |  |  |  |  |
| 769,53 | 769,502509 | 769,5024 | 0,14165 | 303,22 | 303,232954 | 42,72146 | FA 20:4(+O) | PG 36:4 | [M-H]- |
|  |  |  |  | 279,23 | 279,232900 | 10,38570 | FA 18:2 |  |  |
|  |  |  |  | 255,23 | 255,232954 | 11,57387 | FA 16:0(+O) |  |  |
|  |  |  |  | 152,99 | 152,995834 | 38,13321 | PG(153) |  |  |
|  |  |  |  |  |  |  |  |  |  |
| 790,56 | 790,539229 | 790,5391 | 0,16318 | 500,26 | 500,278263 | 36,50702 | -FA 20:2(-H) | PE 40:6 | [M-H]- |
|  |  |  |  | 480,29 | 480,309563 | 40,73164 | -FA 22:6(-H) |  |  |
|  |  |  |  | 303,22 | 303,232954 | 42,72146 | FA 20:4(+O) |  |  |
|  |  |  |  | 283,26 | 283,264254 | 15,01800 | FA 18:0(+O) |  |  |
|  |  |  |  |  |  |  |  |  |  |
| 795,55 | 795,518159 | 795,5182 | -0,05154 | 303,22 | 303,232954 | 42,72146 | FA 20:4(+O) | PG 38:5 | [M-H]- |
|  |  |  |  | 283,25 | 283,264254 | 50,32304 | FA 18:0(+O) |  |  |
|  |  |  |  | 281,24 | 281,248604 | 30,59309 | FA 18:1(+O) |  |  |
|  |  |  |  | 152,99 | 152,995834 | 38,13321 | PG(153) |  |  |
|  |  |  |  |  |  |  |  |  |  |
| 881,54 | 881,518553 | 881,5184 | 0,17356 | 480,29 | 480,309563 | 40,73164 | -FA 22:6(-H) | PI (16:0_22:6) | [M-H]- |
|  |  |  |  | 255,23 | 255,232954 | 11,57387 | FA 16:0(+O) |  |  |
|  |  |  |  | 241,00 | 241,011878 | 49,28631 | PI(241) |  |  |
|  |  |  |  | 223,00 | 223,001313 | 5,88789 | PI(223) |  |  |

**Figure S1** - Loading plot from the major components of the pLSA in the range m/z 600-1050 for the liver groups (C, HC, HT). The identified lipids described in this paper are highlighted. Component 4 is mainly representative for the Heme group. Component 11 is mainly representative for PE(16:0_18:2), PE(18:0_20:4), PG36:4, PE(18:0_22:4). Component 12 and 13 are mainly representative from all the PI.
